# How should we manage abemaciclib in the peri-operative period during secondary breast reconstruction: balancing oncologic benefit and surgical safety

**DOI:** 10.1016/j.breast.2025.104683

**Published:** 2025-12-22

**Authors:** Michel Gabriel Cazenave, Leslie Elahi, Katia Mahiou, Claire Bonneau, Ornella Ammendola, Louise Benoit

**Affiliations:** a Department of Surgical Oncology, Institut Curie, University of Versailles Saint-Quentin en Yvelines, Paris, France; b INSERM U900, Statistical Methods for Precision Medicine, Institut Curie, Saint-Cloud, France

**Keywords:** Abemaciclib, Breast cancer, Breast reconstruction

## Abstract

Abemaciclib, a CDK4/6 inhibitor, has emerged as a pivotal therapy in hormone receptor–positive, HER2-negative breast cancer, including in the adjuvant setting for high-risk early disease. Its integration into long-term oncologic strategies poses new challenges for peri-operative management, particularly in the context of secondary breast reconstruction. Preclinical studies suggest impaired wound healing potential, while clinical data highlight an increased risk of venous thromboembolism. No study to date has specifically addressed surgical outcomes under abemaciclib therapy. We call for the need of a clinical trial in abemaciclib perioperative management and propose pragmatic peri-operative strategies to optimize patient safety without compromising oncologic benefit.

## 1 Introduction

The advent of cyclin-dependent kinase 4/6 (CDK4/6) inhibitors has profoundly modified the therapeutic landscape of hormone-receptor–positive, HER2-negative breast cancer. Among these agents, abemaciclib (LY2835219) occupies a unique position: unlike palbociclib or ribociclib, it is used not only in metastatic disease but also as an adjuvant treatment in early high-risk breast cancer, in combination with endocrine therapy (monarchE trial) [1,2]. It is currently indicated in the adjuvant setting in early-stage hormone-receptor–positive, HER2-negative breast cancer with positive nodes stage N2 or, N1 with either grade 3 cancer or T3 tumor stage for two years. Inevitably, this has confronted surgeons, oncologists and anesthesiologists with a new challenge: how to safely manage abemaciclib in the peri-operative setting, particularly during complex secondary reconstructive procedures such as autologous breast reconstruction. Secondary breast reconstruction includes implant-based, autologous free-flap (e.g., DIEP), and staged procedures, each carrying distinct perioperative risks, particularly regarding wound healing and thrombotic events under abemaciclib.

**Table 1:** Proposed perioperative management strategy for abemaciclib in secondary breast reconstruction.

Domain	Recommendation
Pre-operative Abemaciclib Management	Suspend abemaciclib 5–7 days before surgery
Endocrine Therapy	Continue endocrine therapy
Post-operative Abemaciclib Management	Resume abemaciclib 10–14 days post-op
Thromboprophylaxis	Enhanced VTE prophylaxis
Multidisciplinary Coordination	Team-based planning
Patient Counseling	Educate about risks, interruption rationale

## 2 Biological rationale for concern

CDK4 and CDK6 promote progression from G_1_ phase to S phase of the cell cycle and are essential for regulation of cell proliferation Abemaciclib causes a decrease in phosphorylation of Rb, arrest at G1, and a decrease in cell proliferation [3,4]. *In vitro* studies demonstrate impaired cellular proliferation and migration, raising the theoretical risk of delayed wound healing. Furthermore, abemaciclib has been consistently associated with an increased incidence of venous thromboembolic events (VTE) [5]. Both impaired tissue repair and hypercoagulability represent critical concerns in secondary breast reconstruction. Even more in microsurgical reconstruction, where flap survival hinges on efficient vascular anastomoses and robust tissue integration. Surgical safety would warrant discontinuation of the treatment during the perioperative period.

Yet, the efficacy of abemaciclib lies on the continuation of the treatment for two years post primary surgery. However, due to the psychological effects of a total mastectomy without reconstruction, most patients undergo secondary breast reconstruction before 2 years [6].

## 3 Effects on scarring

LY2835219 is an inhibitor of Rb phosphorylation, which induces a complete cell cycle arrest and suppresses expression of several Rb-E2F-regulated proteins 24 h after a single dose. It inhibits phosphorylation of Rb by CDK4/6 causing a G1 arrest [3]. These findings have been found only in cancer cell lines and cell lines with RB1 mutation or loss are insensitive to the effects of abemaciclib [4]. In epidermal keratinocytes a decrease in cell proliferation was noted and it can be inferred that abemaciclib could affect scarring [7].

However, one recent work studied implant based secondary reconstruction in patients under abemaciclib after radiation therapy. Seventy five patients were included (15 under abemaciclib) and no increase in adverse effects were found [8] To the best of our knowledge, no other clinical work had studied surgical complications linked to abemaciclib.

## 4 Venous thromboembolic events

In the MONARCHE trial (adjuvant abemaciclib in high risk HR + HER2-breast cancer), VTE were more frequent with abemaciclib plus endocrine therapy (2.3 %) versus endocrine therapy alone (0.5 %) [1]. In MONARCH 3 (abemaciclib for advanced BC), VTEs occurred in 4.9 % of patients in the abemaciclib arm versus 0.6 % in the placebo arm [9]. In MONARCH-2 (fulvestrant – abemaciclib versus abemaciclib alone in advanced breast cancer how had progressed under endocrine therapy), thromboembolic occurred in nine patients (2.0 %) in the abemaciclib arm and one patient (0.4 %) in the placebo arm. Of the patients in the abemaciclib arm, four experienced a pulmonary embolism, none of which resulted in death [10]. These works did not study the perioperative period specifically.

In this setting, abemaciclib must be used with thromboprophylaxis and close patient monitoring during surgery.

## 5 Oncological outcomes of treatment discontinuation

Abemaciclib had shown an improved survival in high-risk patients. Since abemaciclib could cause delayed wound healing and VTE, can it be safely discontinued in the perioperative period in case of a secondary breast reconstruction?

*In vitro*, the decrease in pRb and cell cycle progression were sustainable with continuous exposure of breast cancer cells to abemaciclib, with increased effects observed with longer treatment times [4] *In vivo*, target inhibition studies showed LY2835219 produced a cell cycle arrest just 24 h after dosing, and was reversible [3].

In the neo adjuvant clinical setting, abemaciclib, alone or in combination with anastrozole, achieved a significant decrease in Ki67 expression and led to potent cell-cycle arrest after 2 weeks of treatment compared with anastrozole alone in HR positive HER2 negative patient with breast cancer [11]. The impact of treatment discontinuation on Ki67 expression was then studied and they found that discontinuing CDK4 and 6 inhibition after 2 weeks of therapy led to proliferation rebound as measured by Ki-67 in the primary tumour after as little as 4 days of drug cessation [11].

Yet, in another, *in vitro* work, treatment with abemaciclib inhibited cell proliferation and led to G1 cell cycle arrest in a concentration-dependent manner, after longer treatment. These changes were maintained after compound removal, in cells treated with abemaciclib suggesting a prolonged effect [4].

## 6 Current evidence gap

To date, only one study has specifically evaluated post-operative outcomes in patients receiving abemaciclib and found reassuring results on a small cohort. Reports of impaired wound healing remain preclinical, and the safety profile of abemaciclib in surgical oncology remains inferred rather than demonstrated. This lack of data leaves peri-operative decision-making reliant on extrapolation, expert consensus, and individualized risk assessment. Secondary breast reconstruction, especially with free flaps such as the deep inferior epigastric perforator (DIEP) flap, represents a high-risk scenario: large surface areas, microvascular anastomoses, and patients often pre-treated with chemotherapy, radiotherapy, or endocrine therapy. In this context, any factor that might impair cellular repair or increase thrombosis may jeopardize surgical success.

Given these risks, we propose these 3 principles during secondary breast reconstruction, notably for DIEPs (Table 1) [12].

- *Temporary suspension of abemaciclib* – Discontinue abemaciclib 5–7 days before surgery, reflecting its pharmacokinetic half-life. Resume therapy only after satisfactory early wound healing, typically 10–14 days post-operatively.
- *Continuation of endocrine therapy*
- *Enhanced thromboprophylaxis* – Given the elevated thrombotic risk under abemaciclib, peri-operative VTE prophylaxis should be extended and these patients such be considered as high risk following institutional protocols.

Given its 18–28 h elimination half-life, suspending abemaciclib 5–7 days pre-operatively should allow adequate clearance and biologic recovery of CDK4/6-dependent pathways. This would be the time to achieve substantial reduction in steady-state plasma concentration. This is, however, and extrapolation and no consensus exists to date.

Short peri-operative interruptions (≤2 weeks) appear oncologically acceptable in most adjuvant HR+/HER2-high-risk contexts. We would advise caution in very high risks situation such as N2-3 patients or younger patients. It must be reminded that while Ki-67 rebound has been observed as early as 4 days after discontinuation, there is no evidence that such rebound translates into worse survival outcomes during short, clinically necessary interruptions. No clinical data exists to date.

These recommendations may apply differentially depending on surgical complexity and expected wound-healing requirements. While the theoretical risks of delayed wound healing and thrombosis are biologically and clinically plausible, the absence of prospective evidence should not be conflated with reassurance. We propose a pragmatic approach balancing oncologic efficacy with surgical feasibility. Future research should address this knowledge gap through prospective registries and peri-operative outcome studies. Until such data emerge, multidisciplinary dialogue and individualized planning remain the cornerstones of safe care.

## CRediT authorship contribution statement

**Michel Gabriel Cazenave:** Writing – original draft, Methodology, Conceptualization. **Leslie Elahi:** Writing – review & editing, Conceptualization. **Katia Mahiou:** Writing – review & editing, Methodology. **Claire Bonneau:** Writing – review & editing, Methodology. **Ornella Ammendola:** Writing – review & editing, Methodology. **Louise Benoit:** Writing – original draft, Supervision, Methodology, Investigation, Conceptualization.

## Fundings

None.

## Declaration of competing interest

The authors declare that they have no known competing financial interests or personal relationships that could have appeared to influence the work reported in this paper.
